# Motivation for smoking cessation among drug-using smokers under methadone maintenance treatment in Vietnam

**DOI:** 10.1186/s12954-015-0085-7

**Published:** 2015-10-30

**Authors:** Bach Xuan Tran, Long Hoang Nguyen, Huyen Phuc Do, Nhung Phuong Thi Nguyen, Huong Thu Thi Phan, Michael Dunne, Carl Latkin

**Affiliations:** Institute for Preventive Medicine and Public Health, Hanoi Medical University, Hanoi, Vietnam; Johns Hopkins Bloomberg School of Public Health, Baltimore, MD USA; School of Medicine and Pharmacy, Vietnam National University, Hanoi, Vietnam; School of Public Health and Social Work, The Queensland University of Technology, Brisbane, Australia; Hanoi University of Pharmacy, Hanoi, Vietnam; Authority of HIV/AIDS Control, Ministry of Health, Hanoi, Vietnam

**Keywords:** Smoking, Cessation, Stage, Change, Methadone, MMT, Drug use, Vietnam

## Abstract

**Background:**

Smoking cessation treatment service is concerned to be a critical element in methadone maintenance treatment (MMT) in order to diminish the effect of smoke on health outcomes. To implement the smoking cessation services in Vietnam, we examined the stages of change to quit and determined associated factors among MMT patients.

**Methods:**

We conducted a cross-sectional survey with 1016 MMT patients in five clinics in Hanoi and Nam Dinh province, of those, 932 (91.7 %) were ever-smokers. Patients were classified into four groups: “pre-contemplation,” “contemplation,” “preparation,” and “action and maintenance” by using the transtheoretical model. Multivariate logistic regression was applied to determine the associated factor for intention and action to quit smoking.

**Results:**

Overall, 96 % were not actively trying to quit or maintain abstinence. Age older than 45, HIV-positive status, and residence in Hanoi were negatively associated with intention to quit. Meanwhile, higher levels of nicotine dependence and number of years of smoking negatively associated with quitting and abstinence.

**Conclusions:**

The study indicated the high rate of MMT smokers being in pre-contemplation stage but low proportion of quitting and maintaining abstinence. It emphasizes the importance of availability and accessibility of information about smoking cessation therapies and services. Integrating cessation programs into health-care services should be considered to provide tailored interventions for different patient groups.

## Background

Improving health status and supporting healthy behaviors are the goals of interventions for people who inject drugs (PWID) [1]. As a country experienced a rapid spread of HIV infection in drug-using populations, Vietnam has been implementing a comprehensive harm reduction strategy [2]. First introduced in 2008, methadone maintenance treatment (MMT) service has become an essential component of the National HIV/AIDS Strategy [2]. Previous studies have shown that MMT is cost-effective in preventing new HIV cases, improving HIV/AIDS treatment outcomes, and relieving the economic burden of HIV/AIDS and substance abuse on both health systems and affected households [3–5]. MMT, therefore, is the primary intervention that engages IDU into harm reduction programs and health-care services.

Although drug-use behaviors significantly changed over the course of MMT, other unhealthy behaviors may result in diminished health status and quality of life in this patient group [6]. Despite decreasing prevalence of smoking in general population (56 %), it remains high among MMT patients (from 71 to 98 %) [7–10]. In developed countries, some MMT programs have instituted smoking bans, which has reduced the proportion of staff who smoke but not proportion of patients [11]. Evidence showed that an interaction between methadone and nicotine might increase euphoria and diminish mental problems such as restlessness, irritability, and depression [12], by which maintains smoking and reduces patients’ attempts to quit [7]. This interaction is considered a major cause of morbidity, mortality, disability, and poorer quality of life among opioid-dependent smokers compared to non-smokers [13–15]. Therefore, attention should be paid on smoking among PWID, and cessation interventions should be encouraged during MMT [15–18].

Understanding motivation to quit smoking may help predict success of cessation [19, 20]. This is more important among drug users who reported much lower success rate in quitting smoking (12–22 %) than general population (48–58 %) [7–9, 21–23]. Previous studies have shown a number of individuals and environmental factors that influence the process of adapting and maintaining smoking abstinence among PWID. These factors included socioeconomic status, mental health problems, unavailability of health services, family supports, peer influences, and cultural acceptability [23, 24]. Given its high variation across settings, empirical evidence of contextual factors are necessary to design effective smoking cessation programs for PWID.

The rapid expansion of MMT services in Vietnam has provided the treatment to over 30,000 patients [25, 26]. Although the prevalence of smoking among men in Vietnam was high, however, understanding of smoking and motivation to quit among MMT patients are still limited. In this study, we sought to assess patients’ motivation to quit smoking and its associated factors over the course of MMT in a multi-site survey.

## Methods

### Study design and sampling technique

A cross-sectional survey was conducted during January to August 2013 in two northern provinces: Hanoi and Nam Dinh. Five MMT clinics were purposely selected including (1) provincial and district sites, (2) in both rural and urban areas, and (3) different integrative models (Table 1). We invited all patients who registered for MMT at the selected sites to participate in the survey. Eligibility criteria for recruiting participants included (1) taking or initiating MMT in selected sites, (2) presenting at clinics during study period, (3) being 18 years old or above, (4) having capacity to answer questionnaire, and (5) agreeing to participate. A total of 1016 patients (91.5 % response rate) were interviewed, of those, 932 (91.7 %) reported ever smoked that formed the subgroup of this analysis. In a designated room, face-to-face interviews were carried out by well-trained investigators using a structured questionnaire for about 30 min.

**Table 1:** Study settings and sample size

Level	Settings	Site Name	Type of services	Sample size
District (rural)	Nam Dinh City	Provincial AIDS Centre (PAC)	MMT + VCT	248
District (rural)	Xuan Truong District	District Health Centre (DHC)	MMT + VCT + ART + GH	128
District (urban)	Tu Liem District	District Health Centre	MMT + VCT + ART + GH	161
District (urban)	Long Bien District	District Health Centre	MMT + VCT + ART + GH	170
District (urban)	Ha Dong District	Regional Polyclinic (RPC)	MMT + GH	196

*VCT* voluntary HIV testing and counseling, *ART* antiretroviral treatment, *GH* general healthcare

### Measurements

Variables of interest were selected by adopting the socio-contextual model of Sorensen et al. for reducing tobacco use among blue-collar workers [27]. In this study, we measured the influence of patient- and provider-related factors on motivation and readiness to quit smoking among MMT patients.

#### Socioeconomic status

Socioeconomic factors including age, gender, marital status, education, occupation, religion, and income were investigated. Income per month per capita was computed by summing all monthly income sources of household, then dividing to the number of household’s members.

#### Health-related quality of life

Health status was measured using EQ-5D-5L instrument which showed good measurement properties in Vietnamese settings [28]. EQ-5D-5L contains five dimensions (mobility, self-care, usual activities, pain/discomfort, and anxiety/depression) with five response levels [29]. In addition, body mass index and HIV status were recorded.

#### Health-care and MMT service utilization

The use of inpatient and outpatient health services over the past 12 months and duration on MMT were self-reported.

#### Substance abuse

To assess alcohol use, we employed a brief version of the alcohol use disorders identification test-consumption (AUDIT-C) instrument [30]. The Vietnamese version has been used in previous studies [31, 32]. It is comprised of questions with a total score of 0–10. Higher scores indicate higher risks of alcohol dependence. Hazardous drinkers are identified with the threshold of 4 or above for men and 3 or above for women [30, 33]. Additionally, binge drinkers are determined if the respondents have any positive response to the third question. Illicit drug use behaviors included history and current opioid use, duration of drug use, and the experience of previous drug treatment.

#### Smoking-related characteristics

To understand the motivation to quit smoking of MMT patients, we applied the transtheoretical model that describes the progressing of health behaviors through a series of five sequential stages of change. This includes (1) pre-contemplation → (2) contemplation → (3) preparation → (4) action → (5) maintenance. We asked the patients a question: “Are you thinking about quitting smoking?” with four response levels: “No thought of quitting,” “Think I should quit but not quite ready,” “Starting to think about how to change my smoking behavior,” and “Take action to quit” corresponding to the stages of (1) → (4). The stage (5) maintenance included those who have been abstinent of smoking over 6 months.

In addition, the Fagerström test for nicotine dependence (FTND) was used to assess the levels of nicotine dependence among patients. This instrument contains six items that yield a total score of 0–10. Higher score indicates higher level of dependence [34]. Based on the score, patients were classified into following groups: 0–2: very low, 3–4: low, 5: moderate, 6–7: high, and 8–10: very high. Other indicators, including smoking duration, expenses, and number of cigarettes per days, were also recorded. In addition, we asked patients who thought of quitting or cutting down the number of cigarettes they smoke what measures or supports they would like to receive to take action and maintain abstinence.

### Data analysis

The *p* value <0.05 was considered statistically significance. *T* test, ANOVA test, and *χ* [2] were used to measure the difference between means and proportions. To identify the determinants of intention and taking action to quit smoking, we employed multivariate binominal logistic regression, with fractional polynomial model for duration of MMT treatment, to assess the non-linear relationships among variables. In addition, backward stepwise model was approached to include variables with the threshold of *p* values of log-likelihood ratio test <0.2. We then displayed the results by odd ratios (OR) with corresponding 95 % CI. Data analysis was performed by using STATA software version 12.0 (Stata Corp. LP, College Station, United States of America).

### Ethical approval

This study was approved by the Vietnam Authority of HIV/AIDS Control’s Scientific Research Committee. Written informed consents were collected before starting the interview. Respondents could withdraw from the study at any time. The information of patients was coded to ensure confidentiality.

## Results

Of 932 respondents, the mean age of sample was 36.5 years (SD = 7.4). The predominant groups were male (98.8 %), with a religious orientation of cult of ancestors (88.7 %), and living with spouse/partner (66.7 %). Most of the respondents attained less than high school (54.3 %) and had employment (74.3 %). The majority of the participants had income more than 2.5 million VND per month (43.6 %) (Table 2). Table 2 also shows the health status and health-care utilization of patients. There were 7.9 % of respondents who were HIV-positive and 6.3 % currently taking ART medication. Based on the EQ-5D-5L, about one fifth of sample reported anxiety/depression (20.2 %) and 17.1 % felt pain/discomfort. About 22 % had used outpatient health-care service in the last 12 months, while 8.3 % used inpatient services.

**Table 2:** Demographic, health status, and health-care utilization of respondents

	Pre-contemplatio*n*		Contemplatio*n*		Preparation		Action and Maintenance		Total		*p*
	*n*	%	*n*	%	*n*	%	*n*	%	*n*	%	
Total	477	51.2	212	22.8	206	22.1	37	4.0	932	100.0	
	Mean	SD	Mean	SD	Mean	SD	Mean	SD	Mean	SD	
Age	37.0	7.7	36.1	7.3	36.0	6.7	35.3	7.0	36.5	7.4	0.19
Sex (male)	473	99.2	212	100.0	202	98.1	34	91.9	921	98.8	<0.01
Marital status											
Single/divorced/widow	160	33.5	61	28.8	78	37.9	11	29.7	310	33.3	0.25
Live with spouse/partner	317	66.5	151	71.2	128	62.1	26	70.3	622	66.7	
Educational attainment											
<High school	277	58.1	114	54.0	91	44.4	23	62.2	505	54.3	<0.01
High school	177	37.1	85	40.3	90	43.9	12	32.4	364	39.1	
>High school	23	4.8	12	5.7	24	11.7	2	5.4	61	6.6	
Employment											
Unemployed	131	27.5	59	27.8	39	18.9	11	29.7	240	25.8	0.09
Currently working	346	72.5	153	72.2	167	81.1	26	70.3	692	74.3	
Religion											
Cult of ancestors	428	89.7	188	88.7	180	87.4	31	83.8	827	88.7	0.62
Others	49	10.3	24	11.3	26	12.6	6	16.2	105	11.3	
Income per capita											
<1.2 million VND	101	21.2	48	22.6	38	18.5	7	18.9	194	20.8	0.55
1.2–2.5 million VND	160	33.5	72	34.0	86	41.8	14	37.8	332	35.6	
>2.5 million VND	216	45.3	92	43.4	82	39.8	16	43.2	406	43.6	
Location											
Nam Dinh	313	65.6	117	55.2	104	50.5	14	37.8	548	58.8	<0.01
Hanoi	164	34.4	95	44.8	102	49.5	23	62.2	384	41.2	
Area of clinics											
Rural	60	12.6	32	15.1	31	15.1	8	21.6	131	14.1	0.40
Urban	417	87.4	180	84.9	175	85.0	29	78.4	801	85.9	
MMT model											
MMT + VCT	104	21.8	63	29.7	71	34.5	15	40.5	253	27.2	<0.01
MMT + GH	111	23.3	34	16.0	50	24.3	5	13.5	200	21.5	
MMT + VCT + GH + ART	262	54.9	115	54.3	85	41.3	17	46.0	479	51.4	
Health-related status											
Body mass index											
Normal weight	366	76.7	151	71.2	152	73.8	28	75.7	697	74.8	0.57
Underweight	28	5.9	19	9.0	13	6.3	4	10.8	64	6.9	
Overweight, obesity	83	17.4	42	19.8	41	19.9	5	13.5	171	18.4	
HIV-positive	47	9.9	10	4.7	12	5.8	5	13.5	74	7.9	0.04
Current taking ART	36	7.6	9	4.3	10	4.9	4	10.8	59	6.3	0.42
Currently feeling pain	83	17.4	32	15.1	40	19.4	4	10.8	159	17.1	0.49
Currently feeling anxiety/depression	89	18.7	35	16.5	57	27.7	7	18.9	188	20.2	0.02
Health-care services utilization in the last 12 month											
Outpatient service utilization	85	17.8	57	26.9	53	25.7	11	29.7	206	22.1	0.01
Inpatient service utilization	43	9.0	19	9.0	14	6.8	1	2.7	77	8.3	0.46

Table 3 indicates substance-use behaviors among MMT patients. More than a half of respondents smoked over 10 cigarettes per day. The proportions of people smoking within 5 min of waking and even if sick in bed were 32.8 and 0.9 %, respectively. About half of respondents reported a moderate to very high levels of nicotine dependence. The mean age of initial smoking were 17.2 (SD = 3.5), and the mean duration of smoking was 14.1 years (SD = 8.5). On average, participants spent 300 thousand Vietnam dong (~USD 15, 2013 exchange rate) monthly for tobacco.

**Table 3:** Smoking, nicotine dependence and other substance abuse

	Pre-contemplation		Contemplation		Preparation		Action and maintenance		Total		*p*
	*n*	%	*n*	%	*n*	%	*n*	%	*n*	%	
Smoking											
Number of cigarettes per day											
≤10	215	46.9	101	47.6	85	41.7	13	59.1	414	46.2	0.64
≥11–20	206	45.0	99	46.7	98	48.0	8	36.4	411	45.9	
21–30	24	5.2	8	3.8	16	7.8	1	4.6	49	5.5	
>30	13	2.8	4	1.9	5	2.5	0	0.0	22	2.5	
Smoke within 5 min of waking	156	33.7	57	27.0	79	38.4	9	24.3	301	32.8	0.13
Smoke even if sick in bed	146	32.3	68	32.7	57	28.1	7	19.4	278	30.9	0.30
Nicotine dependence level											
Very low	129	27.0	64	30.2	49	23.8	21	56.8	263	28.2	<0.01
Low	130	27.3	51	24.1	58	28.2	7	18.9	246	26.4	
Moderate	65	13.6	29	13.7	23	11.2	2	5.4	119	12.8	
High	93	19.5	53	25.0	36	17.5	5	13.5	187	20.1	
Very high	60	12.6	15	7.1	40	19.4	2	5.4	117	12.6	
	Mean	SD	Mean	SD	Mean	SD	Mean	SD	Mean	SD	
Age at smoking initiation	17.02	3.4	17.05	3.7	17.87	3.4	17.49	2.9	17.24	3.5	0.02
Duration of regular smoking in years	14.41	8.9	13.15	8.3	14.88	7.7	10.49	7.6	14.07	8.5	<0.01
Expense for smoking (thousand VND per month)	302.26	312.5	289.59	272.5	310.03	259.1	140.59	185.2	294.68	289.6	<0.01
FTND score	4.36	2.5	4.22	2.3	4.67	2.6	2.89	2.5	4.34	2.5	<0.01
Drug use											
Ever inject drug	369	77.4	143	67.5	160	77.7	24	64.9	696	74.7	0.02
Current drug use	24	5.0	8	3.8	12	5.8	1	2.7	45	4.8	0.71
# drug rehabilitation											
None	35	7.3	17	8.0	15	7.3	2	5.4	69	7.4	0.71
1–5 episodes	307	64.4	147	69.3	133	64.6	28	75.7	615	66.0	
6–10	108	22.6	36	17.0	47	22.8	4	10.8	195	20.9	
>10	27	5.7	12	5.7	11	5.3	3	8.1	53	5.7	
	Mean	SD	Mean	SD	Mean	SD	Mean	SD	Mean	SD	
Age at first drug use	24.49	6.6	24.18	7.0	24.32	6.1	24.24	6.0	24.37	6.5	0.95
Age at first drug injection	27.06	7.6	26.13	6.6	26.94	7.1	25.25	5.4	26.78	7.2	0.41
Time since 1st drug use	13.55	5.9	12.88	5.9	12.70	5.2	12.03	5.3	13.15	5.7	0.14
Time since 1st drug injection	10.40	5.0	9.99	4.9	9.69	4.8	10.00	4.0	10.14	4.9	0.46
Duration of MMT (month)	17.10	11.0	16.83	11.2	14.57	10.0	13.56	9.3	16.34	10.8	0.02
Alcohol use											
Hazard Drinking (*n* = 480)	130	56.3	72	59.0	62	56.9	10	55.6	274	57.1	0.97
Binge Drinking (*n* = 480)	124	53.7	63	51.6	59	54.1	11	61.1	257	53.5	0.89

Table 3 also shows that the mean age of initial drug use was 24.4 years (SD = 6.5). There were 74.7 % respondents ever injected drug, and 4.8 % of patients were concurrently using opiates during MMT. Most of the samples had one to five episodes of drug rehabilitation (66.0 %). As for alcohol use, the prevalence of hazard and binge drinking was 57.1 and 53.5 %, respectively.

There was only 4 % currently taking actions to quit smoking, meanwhile 22.1 % were in preparation stage and 22.8 % were in contemplation stage where they thought of quitting. In Table 4, preference for cessation supports were explored among those who were aware of the harms of smoking (*n* = 455). Of 423 responses (93 %), self-administration without others’ supports was the most preferable approach for smoking cessation among MMT smokers (77.6 %), followed by using nicotine replacement therapy (11.3 %) and having familial (5 %) and health workers’ (4.5 %) supports.

**Table 4:** Preference for support among MMT patients who thought of smoking cessation

Cessation support	Number	Percent
Health staff support	19	4.5
Family support	21	5.0
Friend/peer support	6	1.4
Using nicotine replacement therapy	48	11.3
Using herbs	2	0.5
Using acupuncture	1	0.2
Use mobile phone	1	0.2
Self-help	325	76.8

Results from reduced multivariate logistic regression are shown in Table 5. The intention to quit smoking of MMT patients was negatively associated with age older than 45, HIV-positive status, and residence in Hanoi, while positively associated with having outpatient health-care services in the last 12 months. As for quitting and maintenance, we found that the level of nicotine dependence and longer years of smoking negatively predicted quitting and maintaining abstinence among MMT patients. In these models, the duration on MMT has been treated as a polynomial factor that showed a negative association between the number of months on MMT and the likelihood of smoking abstinence; however, it was not statistically significant.

**Table 5:** Factors associated with Intention to quit and quitting smoking

	Intention to quit			Quitting smoking		
	OR	95 % CI		OR	95 % CI	
Province (Hanoi vs Nam Dinh)	0.47***	0.34	0.67	0.50*	0.23	1.08
Location (Urban vs Rural)	1.44	0.90	2.31			
Duration of MMT (month)	1.00	0.99	1.02	0.99	0.95	1.03
Outpatient service use last 12 months (yes vs no)	1.98***	1.44	2.72			
# of drug rehabilitation				1.03	0.98	1.07
Religion (vs cult of ancestors)						
Buddhism				2.72*	0.84	8.78
Occupation (vs unemployed)						
Other jobs	1.41	0.87	2.28			
Age (vs 18–<25)						
35–<40				2.63**	1.30	5.30
≥45	0.51***	0.34	0.78			
Marital status (vs single)						
Live with spouse	1.23	0.91	1.67			
Income quintile (vs poorest)						
Rich	0.78	0.56	1.09			
Having pain/discomfort (yes vs no)				0.41	0.12	1.43
HIV status (vs negative)						
Positive	0.58**	0.34	0.99			
Body mass index (vs underweight)						
Normal	1.47	0.86	2.52			
Level of nicotine dependence (vs very low)						
Low				0.31**	0.12	0.81
Moderate				0.22**	0.05	0.97
High				0.37**	0.13	1.04
Very high				0.22**	0.05	0.99
Year of smoking				0.95**	0.91	1.00

*p < 0.1; **p < 0.05; *** *p* < 0.01

## Discussion

This is the first study investigating factors associated with the motivation of MMT patients to quit smoking in Vietnam. Using the transtheoretical model, we characterized the stages that MMT patients are in with regard to their smoking behaviors. The findings showed a substantial proportion of MMT patients were at the pre-contemplation stage. They did not have any intention to change their smoking behaviors. It is important to note that intentions to quit and taking action to quit smoking were not improved over the course of MMT.

In this study, 44.9 % respondents were in the contemplation and preparation stages and only 4.0 % took action to quit smoking and maintained abstinence. These figures were lower than previous studies in MMT patients. For example, a study of Nahvi et al. showed that 48 % of MMT smokers were in contemplation stage and 22 % were in preparation stage [9]. Another study of Richter et al. suggested that 46 % of MMT patients were in contemplation stage [35]. Since having an intention to quit smoking may predict successful smoking abstinence [36], the low rate of intention to quit smoking in the present study may reflect the lack of smoking cessation interventions and antismoking campaigns targeting this population. For MMT patients, as illicit drug use is highly stigmatized in Vietnam, it is likely that their focus is on opiate absences and do not view cessation of tobacco use as a priority. Moreover, smoking is normative among men in Vietnam with over half of the adult male population currently smoking.

Literatures documented that patients might suffer from withdrawal problems (stress/anxiety or other mental problems) while attempting to quit smoking [37] due to nicotine dependence [38]. In our study, higher level of nicotine dependence and number of years of smoking were negative predictors of patient’s abstinence to smoking [39, 40]. This is similar to findings by John et al. who also suggested that nicotine dependence may increase the number of quit attempt but decrease the likelihood of abstinence [38]. As for preferred supports for smoking cessation, the majority of respondents chose self-administration while having cessation-related health was very limited. It may be because of the unawareness of smokers and unavailability of smoking cessation services as well as other socioeconomic barriers [41].

Noticeably, the duration of MMT was inversely related to the intention, although this association was not statistical significance. To date, prior studies have not examined this association. Since the duration of MMT treatment was proportionate with the reduction of MMT doses, patients with lower doses of methadone were more likely to report quit intensions [9]. Additional research to investigate the interaction between duration of MMT and MMT doses on readiness to quit smoking is warranted.

This study suggested several implications. First, clinicians should understand MMT patients’ stage of change for smoking cessation in order to implement tailored counseling and interventions. They should first ask about smoking in MMT clients, then ask about their interest in quitting. Patients who report no interest should be counseled using motivational interviewing about dangers of smoking and ability for people to quit. Those who express interest should be provided with a brief smoking cessation intervention, the “five A’s,” including ask, advise, assess, assist, and arrange [42]. These tailored clinical interventions should be coupled with community level interventions [41]. Second, providing smoking cessation treatment by integrating into MMT clinics (on-site program) may encourage the motivation for smoking cessation. Besides, for those smokers taking antiretroviral therapies, intervening on smoking may also improve treatment adherence and outcomes [43]. Finally, capacity of health staffs in terms of screening and counseling should be enhanced by training, which helps to enhance the provision of smoking cessation treatment [41].

The strengths of this study included a large sample of MMT patients in various Vietnamese settings. In addition, we employed several measures (EQ-5D-5L, FTND, AUDIT-C) that showed good measurement properties in these patient groups in Vietnam [3, 28, 31, 32, 44, 45]. Nonetheless, some limitations should be acknowledged. First, causal relationships between motivation to quit and related factors cannot be established due to the cross-sectional design. Second, data collection was based on self-reports, which might lead to recall bias. Finally, some psychosocial factors such as methadone dose, self-efficacy, depression, tobacco availability, social norms, and smoking cost data were not collected, suggesting the further research to investigate those factors in order to better understand the mechanism of smoking behaviors and develop appropriate interventions.

## Conclusions

In conclusion, the study indicated the high rate of MMT smokers being in pre-contemplation stage but low proportion of quitting and abstinence. This study also underlined the importance of availability and accessibility of information about smoking cessation therapies with the high-quality consultation and services. Integrating cessation programs into health-care services should be considered to provide tailored interventions for different patient groups.
